# Accuracy of the Dexcom G6 Glucose Sensor during Aerobic, Resistance, and Interval Exercise in Adults with Type 1 Diabetes

**DOI:** 10.3390/bios10100138

**Published:** 2020-09-29

**Authors:** Florian H. Guillot, Peter G. Jacobs, Leah M. Wilson, Joseph El Youssef, Virginia B. Gabo, Deborah L. Branigan, Nichole S. Tyler, Katrina Ramsey, Michael C. Riddell, Jessica R. Castle

**Affiliations:** 1 Division of Endocrinology, Harold Schnitzer Diabetes Health Center, Oregon Health & Science University, Portland, OR 97239, USA; guillotf@ohsu.edu (F.H.G.); wilsolea@ohsu.edu (L.M.W.); elyoussj@ohsu.edu (J.E.Y.); gabo@ohsu.edu (V.B.G.); branigad@ohsu.edu (D.L.B.); castleje@ohsu.edu (J.R.C.); 2 Artificial Intelligence for Medical Systems Lab, Department of Biomedical Engineering, Oregon Health & Science University, Portland, OR 97239, USA; tylern@ohsu.edu; 3 Oregon Clinical and Translational Research Institute Biostatistics & Design Program, Oregon Health & Science University, Portland, OR 97239, USA; ramseyk@ohsu.edu; 4 Muscle Health Research Centre, School of Kinesiology and Health Science, York University, Toronto, ON M3J 1P3, Canada; mriddell@yorku.ca

**Keywords:** continuous glucose monitoring, type 1 diabetes, exercise, glucose sensor accuracy, high intensity interval training, aerobic exercise, resistance exercise

## Abstract

The accuracy of continuous glucose monitoring (CGM) sensors may be significantly impacted by exercise. We evaluated the impact of three different types of exercise on the accuracy of the Dexcom G6 sensor. Twenty-four adults with type 1 diabetes on multiple daily injections wore a G6 sensor. Participants were randomized to aerobic, resistance, or high intensity interval training (HIIT) exercise. Each participant completed two in-clinic 30-min exercise sessions. The sensors were applied on average 5.3 days prior to the in-clinic visits (range 0.6–9.9). Capillary blood glucose (CBG) measurements with a Contour Next meter were performed before and after exercise as well as every 10 min during exercise. No CGM calibrations were performed. The median absolute relative difference (MARD) and median relative difference (MRD) of the CGM as compared with the reference CBG did not differ significantly from the start of exercise to the end exercise across all exercise types (ranges for aerobic MARD: 8.9 to 13.9% and MRD: −6.4 to 0.5%, resistance MARD: 7.7 to 14.5% and MRD: −8.3 to −2.9%, HIIT MARD: 12.1 to 16.8% and MRD: −14.3 to −9.1%). The accuracy of the no-calibration Dexcom G6 CGM was not significantly impacted by aerobic, resistance, or HIIT exercise.

## 1. Introduction

Regular physical activity can help people with type 1 diabetes improve glycemic control, decrease total daily insulin needs, achieve a healthier body mass index, and reduce risk of cardiovascular and peripheral neuropathy complications [1,2,3]. Despite these benefits, many people living with type 1 diabetes do not engage in the recommended amount of physical activity [4]. Among the many challenges people with type 1 diabetes face when exercising, fear of hypoglycemia has been identified as the strongest barrier [5]. Moderate intensity aerobic exercise, for example, is known to cause sharp drops in glucose levels [6,7]. Conversely, high intensity exercises such as resistance training or high intensity interval training (HIIT) can lead to hyperglycemia [8,9]. Exercise intensity and duration, insulin-on-board, and carbohydrate intake are also known to affect glucose response [10]. A consensus statement was published in 2017 to provide recommendations on how to reduce the risk of exercise-related dysglycemia [1].

The emergence of continuous glucose monitoring (CGM) has transformed how type 1 diabetes is managed and enabled better monitoring of glucose during exercise [11,12]. The use of CGM systems has been shown to improve time-in-range, while reducing HbA1c levels and risk of hypoglycemia [13,14,15,16]. Additionally, it has led to the development of closed-loop systems associated with significant improvements in time-in-range [17]. However, the reliability of past CGM systems during exercise remains in question [18,19]. Among their known challenges, CGM devices tend to be less accurate at extreme values and during periods of rapid changes [20], two common scenarios observed during physical activity. Inherently, these devices provide an indirect estimate that is biased by a physiological time delay associated with the equilibration of glucose between the blood and interstitial fluid compartments [21,22,23]. Exercise further impacts CGM accuracy through its impact on volume and fluid distribution within the interstitial compartment [24] and its stimulation of endogenous glucose production [25].

While several studies have assessed the accuracy of CGM systems during exercise [26,27,28,29], these studies focused predominantly on earlier generations of sensors, which have been shown to be less accurate than currently available products [30,31]. As CGM devices continue to evolve and improve their accuracy, additional studies are required to characterize how well they perform during exercise. Although the general accuracy of the latest factory-calibrated Dexcom G6 system has been examined [32,33], no study has yet assessed the impact that exercise has on the accuracy of this sensor.

With diverse weekly physical activity being recommended for all adults living with diabetes [7], it is critical for healthcare providers and patients to understand the accuracy of CGM during exercise. As such, this analysis aims to evaluate and compare the accuracy of the commercially available Dexcom G6 system during aerobic, resistance, and HIIT exercise in adults with type 1 diabetes with reference to capillary blood glucose levels. Early results of this study were given as an oral presentation at the Advanced Technologies and Treatments for Diabetes, 2020 conference [34].

## 2. Materials and Methods

### 2.1. Study Design and Protocol

This work is a planned secondary analysis of a single center randomized prospective study involving 24 adults with type 1 diabetes using multiple daily injection therapy. This outpatient study aimed to assess the impact of weekly physician-driven insulin setting changes on participants with elevated HbA1c levels of 7.0% or more. Physician’s recommendations were used to improve and compare the performance of an artificial-intelligence-based decision support algorithm as previously published [35]. This clinical trial was approved by the Institutional Review Board at Oregon Health & Science University (OHSU) on 9 March 2018, and additional information can be found at https://clinicaltrials.gov under the identifier NCT03443713. All participants gave informed consent prior to participating in this trial.

Participants wore a commercial Dexcom G6 CGM (Dexcom Inc., San Diego, CA, USA) on their abdomen for 10 days at a time, for a total of 28 days, and were instructed not to perform calibrations. Daytime physical activity was captured using an Apple Watch (Apple Inc., Cupertino, CA, USA). Participants used the InPen device (Companion Medical, San Diego, CA, USA) for administration of insulin aspart and were instructed to use the InPen bolus calculator on a smartphone. They used either insulin glargine or insulin degludec for long-acting insulin. Participants logged all food and drinks consumed during the study using a mobile application developed at OHSU. Each participant was asked to exercise at home four times and to complete two exercise sessions in clinic. The first in-clinic session was scheduled between day 9 and 14 of the study, and the second between day 16 and 20. Both at-home and in-clinic exercise sessions followed standardized videos. Participants were randomly assigned to either an aerobic, resistance, or HIIT exercise video. The 30-min aerobic exercise video was designed for steady aerobic workout and consisted of 45 s of activity interrupted by 15 s of instructional transition. The 30-min resistance exercise video was designed to work all muscle groups and made use of resistance bands with self-selected resistance level. The shorter 20-min HIIT video consisted of 40-s activities followed by 80 s of rest.

During the in-clinic exercise sessions, capillary blood glucose (CBG) was measured using a Contour Next meter (Ascensia Diabetes Care Holdings AG, Basel, Switzerland), which is among the most accurate commercially available glucose meters (mean absolute relative difference (MARD) of 5.6 ± 6.4%) [36]. Capillary blood glucose measurements were collected just before the start of exercise (minute 0), every 10 min until the end of exercise, and 15- and 30-min post-exercise. As such, the evaluated time points for aerobic and resistance exercise were at time 0, 10, 20, 30, 45 and 60 min, while the shorter 20-min HIIT video resulted in time 0, 10, 20, 35 and 50 min. Heart rate data were captured using a Polar H10 heart rate monitor.

Exercise was only initiated once the participant’s glucose was above 80 mg/dL (4.4 mmol/L) with carbohydrates administered for values below 120 mg/dL (6.7 mmol/L) at the start of exercise, or below 70 mg/dL (3.9 mmol/L) thereafter. Serum ketone levels were checked for glucose values above 250 mg/dL (13.9 mmol/L) and the exercise session was rescheduled if the ketone levels were above 0.6 mmol/L.

### 2.2. Statistical Analysis

Capillary glucose measurements from the Contour Next meter were used as reference glucose and were paired with the temporally nearest CGM values within 2.5 min. Only CBG values between 40 and 400 mg/dL (2.2–22.3 mmol/L) were considered, excluding 3 out of 272 m values.

The accuracy of the Dexcom G6 system during exercise was evaluated using median absolute relative difference (ARD) and relative difference (RD), comparing subsequent measures to baseline. Because the paired differences of these measures did not follow a normal distribution, Wilcoxon signed-rank tests were used to determine the statistical significance of the presented results, at a 95% confidence level (alpha = 0.05) [37].

Next, mixed-effects regression models were used to estimate the effects of four independent variables on mean ARD and mean RD. The mean RD was found to reasonably fit a normal distribution. However, the mean ARD data were strictly positive and right-skewed, thus better fitting a gamma distribution. As such, mean RD was estimated using a linear regression model while mean ARD was estimated using a gamma regression model. The independent variables were selected based on hypothesized relationships to the endpoints and include (1) the exercise type (aerobic, resistance or HIIT), (2) the reference CBG value, centered at the mean, (3) the change in CBG over the previous 10-min interval (glucose drop), and (4) the time since exercise start in 10-min increments, starting at 10 min. These variables were then scaled for interpretability. Higher-order terms and splines were considered and tested for some of the models based on exploratory analyses, but these failed to provide a better fit and were not used in the final analysis. Additionally, both regression models also included nested random intercepts for the study participant and the exercise session. Robust (empirical) variance estimators were used to calculate *p*-values from the Wald test of model coefficients and 95% confidence intervals (CI). The gamma model for ARD was estimated using a generalized linear mixed model with a log link. Because mean ARD did not follow a normal distribution, its effects are reported as ratios. Lastly, aerobic exercise was selected as reference in both models, as it represented the greatest change in glucose when compared with resistance and HIIT exercises. In a preliminary analysis, we investigated whether the time since application of the CGM influenced accuracy. We did not find that sensor wear-time was statistically related to accuracy (*p* = 0.201), and since the study was not powered to detect differences in accuracy across wear times, we did not include this variable in the final mixed effects analysis.

The Clarke error grid analysis (EGA) was then used to visualize the data and quantify the clinical accuracy of the system [38]. This analysis is specifically used to assess the possible impact of inaccuracy on diabetes treatment and allows for an estimation of safe and unsafe glucose measurements compared to reference values. Assessment of the statistical significance of the Clarke EGA results was performed using a χ^2^ test [39,40]. Next, a lead-lag analysis was performed to compare the CGM and meter signals through maximization of the cross-correlation coefficient [41]. This lead-lag analysis was conducted by first linearly interpolating the sensor and meter data over 1-min intervals to increase the number of data points available. The sensor data were then shifted back and forth in time by up to 30 min relative to the meter data. Correlation coefficients were calculated for each minute and the highest correlation factor was used to estimate the lead or lag of the sensor. All analyses were completed using MATLAB R2019b (Mathworks, Natick, MA, USA) and Stata/IC version 15.1 (StataCorp., College Station, TX, USA) [42,43].

## 3. Results

A total of 25 participants were enrolled, of which one withdrew prior to any exercise visits due to scheduling conflicts. All remaining 24 participants completed two exercise sessions in clinic and were included in the analysis. Eight persons were randomly assigned to each exercise group (aerobic, resistance and HIIT) for a total of 48 exercise visits and 16 sessions per exercise type. The baseline characteristics and demographics of these participants are reported in Table 1. The CGM sensors were applied on average 5.3 days prior to the in-clinic visits (range 0.6–9.9), with similar wear time across all exercise types and between the first exercise visit (mean 4.3 days, range 0.6–9.9) and second visit (mean 6.3 days, range 3.0–9.2).

In total, 272 paired Dexcom G6 CGM and reference Contour Next CBG values were analyzed: 96 pairs for aerobic, 96 for resistance exercise, and 80 for HIIT. As expected, the sharpest drop in median CBG from the start to the end of exercise was observed during aerobic exercise (−47.5 mg/dL or −2.6 mmol/L), followed closely by resistance exercise (−36.5 mg/dL or −2.0 mmol/L) and HIIT (−26.0 mg/dL or −1.4 mmol/L). Interstitial glucose results from the G6 sensor showed a similar trend with the largest glucose drop during aerobic exercise (−46.5 mg/dL or −2.6 mmol/L), followed by resistance (−20.5 mg/dL or −1.1 mmol/L) and HIIT (−15.5 mg/dL or −0.9 mmol/L). From the start to the end of exercise, the majority (69%) of participants experienced a drop in blood glucose.

**Figure 1:**
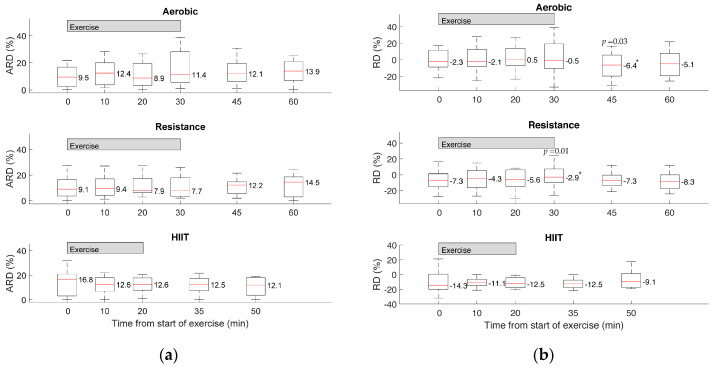
(**a**) Absolute relative differences (ARD, %) and (**b**) relative differences (RD, %) for aerobic, resistance and high intensity interval training (HIIT) exercise. Median values that are statistically significant from baseline are indicated by an asterisk (*), with the corresponding *p*-value listed above.

Median ARD did not differ significantly from the start of exercise to the end of exercise across all exercise types, as illustrated in Figure 1. Median RD demonstrated a negative bias across nearly all sample observations and across each of the exercise types, meaning that the CGM generally reported lower glucose than the Contour Next CBG, including at the start of exercise. Median RD only showed a statistically significant larger negative bias relative to baseline at time 45 min of aerobic exercise (*p* = 0.03). For resistance exercise, the RD at time 30 min was statistically different from baseline (*p* = 0.01), but the negative bias at time 30 min was smaller than baseline. Relative to the start of exercise, the median RD for all other time points did not show any significant changes.

Three independent variables including exercise type, reference CBG value and glucose drop were significantly associated with RD in the mixed-effects linear regression model (Table 2). Time from start of exercise was not significantly associated with mean RD when meter value and glucose drop were included in the regression model. The intercept, representing the mean RD when other variables are null, was very close to zero at −0.2% (*p* = 0.96), meaning that there was no substantial bias in the CGM when controlling for other variables (95% CI −7.2 to 6.8). When evaluating the influence of exercise type on RD, we found that the mean RD was significantly larger in the negative direction during HIIT with a difference of −11.3% compared to aerobic exercise (*p* = 0.04, 95% CI −22.0 to −0.5). In comparison, resistance exercise was found to be somewhat closer to aerobic exercise, with a difference of −4.6% (*p* = 0.39; 95% CI −15.0 to 5.8). Mean RD was also found to be strongly linked with capillary glucose levels with a change of −0.74% for every 10 mg/dL (0.5 mmol/L) increase in the reference blood glucose (*p* = 0.003; 95% CI −1.2 to −0.26). Lastly, mean RD and glucose drop were strongly correlated with a 3.1% change in mean RD for every 10 mg/dL of glucose drop, meaning that as glucose dropped faster, we observed a more positive RD (*p* < 0.001; 95% CI 2.3 to 3.9). Observations from exercise sessions were correlated with an intraclass correlation coefficient (ICC) of 0.78, while the ICC from participants was 0.43.

On the other hand, the regression model for mean ARD did not link changes with any of the independent variables at a statistically significant level. Time from start of exercise was not significantly associated with ARD when meter value and glucose drop were included in the regression model. The estimated effect was 1.03 on the ratio scale for every 10 min (*p* = 0.40; 95% CI 0.97 to 1.1), meaning that a 0.3% increase in mean ARD is predicted for every 10 min of exercise. The intercept, or mean ARD when other variables are null, was significantly different from zero at 10.85% (*p* < 0.001; 95% CI 7.0 to 16.9). Mean ARD during HIIT was nearly the same as during aerobic exercise, with a ratio of 0.98 (*p* = 0.94; 95% CI 0.6 to 1.6). Resistance exercise was somewhat lower at 0.93 (*p* = 0.79; 95% CI 0.6 to 1.5). In practice, these ratios represent a predicted mean ARD of 13.3% (CI 8.4 to 18.2) for aerobic exercise, followed by 13.0% (CI 9.3 to 16.7) for HIIT and 12.4% (CI 8.8 to 15.9) for resistance exercise. ARD changed only fractionally for every 10 mg/dL increase in the reference blood glucose (ratio 0.997; *p* = 0.83; 95% CI 0.97 to 1.02). This represents a small decrease in mean ARD as CBG increases, with predicted ARD ranging from 13.3% (CI 9.3 to 17.3) at 70 mg/dL (3.9 mmol/L) down to 12.6% (CI 9.2 to 16) for glucose levels near 250 mg/dL (13.9 mmol/L). ARD changed little for every 10 mg/dL of glucose drop (ratio 1.03; *p* = 0.31; 95% CI 0.97 to 1.1). That is, mean ARD is predicted to rise from 12.7% (CI 10.5 to 14.8) at constant glucose levels up to 14.0% (CI 11.1 to 17.0) for glucose drops of 30 mg/dL every 10 min.

**Figure 2:**
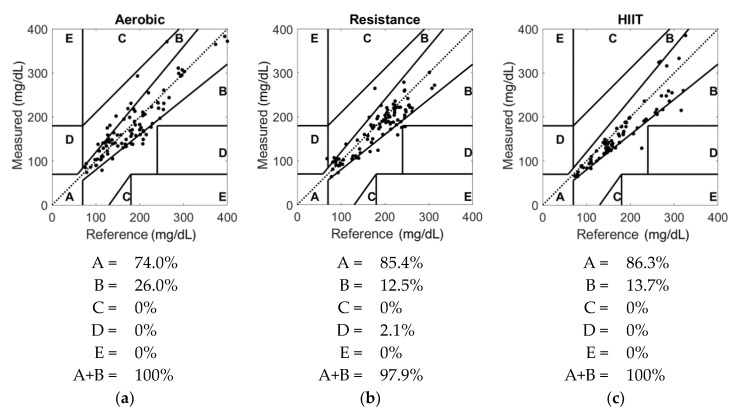
Clarke error grid analysis showing paired continuous glucose monitoring (CGM) and capillary (reference) glucose during (**a**) aerobic, (**b**) resistance and (**c**) high intensity interval training (HIIT) exercise. In these scatterplots, the diagonal represents perfect agreement between capillary and sensed glucose. The regions (or zones) labelled A through E represent varying degrees of accuracy of glucose estimations. Zone A contains values within 20% of the reference sensor. Zone B values deviate more than 20% but would not lead to inappropriate treatment. Values within zone C would lead to overcorrecting while those in zone D represent a potentially “dangerous failure to detect and treat”. Lastly, zone E would result in opposite treatment decisions (e.g., treatment of hypoglycemia for hyperglycemia and vice versa) [38].

The Clarke error grid analysis (EGA) results for the different types of exercises are shown in Figure 2. Although the accuracy drop in the A-region between aerobic exercise and HIIT was found to be significant (*p* = 0.04), the bias in the clinically safe zone (A+B) was comparable across exercise types. Additionally, no notable differences were observed in these metrics when comparing baseline values (before exercise) to those at different time points during exercise.

**Figure 3:**
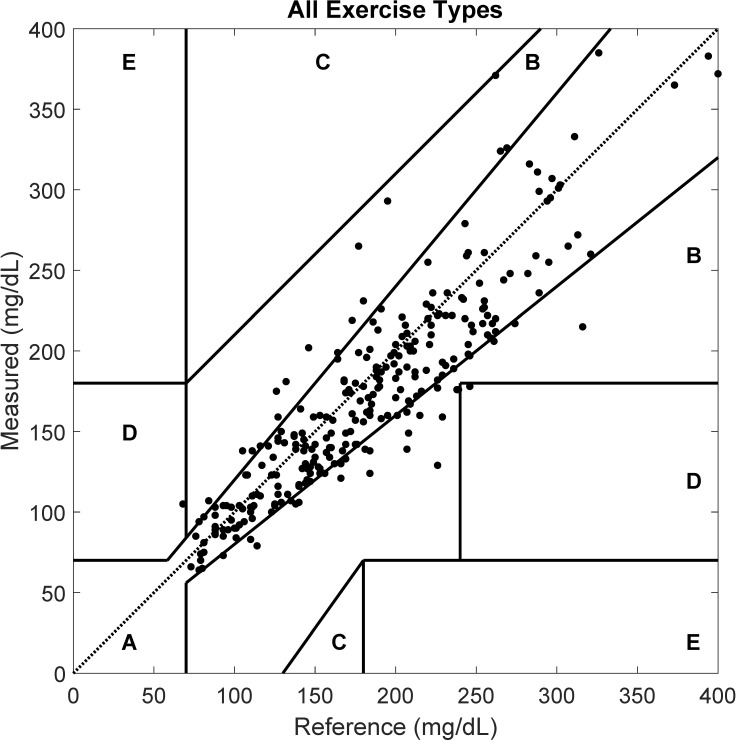
Clarke error grid analysis showing paired CGM and capillary (reference) glucose for all exercise types combined.

Lastly, the Clarke EGA shown in Figure 3 effectively represents the overall accuracy of the Dexcom G6 sensor during all exercise types, with the vast majority of the collected data (99.3%) located in the clinically safe regions (A+B).

The lead-lag analysis for all exercises combined resulted in a median estimated lag of 13 min (interquartile range (IQR): −25.5 to 29.5 min). Lag appeared to be greater for resistance and HIIT exercise with a median of 18 min (IQR −26.5 to 30 min) and 19 min (IQR 4.5 to 29 min), respectively, compared to a 1-min (IQR: −26.5 to 21 min) estimated lag for aerobic exercise. Visually, however, lag was not apparent in many of the individual glucose traces.

Throughout this study, a total of three adverse events occurred that were deemed unrelated to the study. One additional adverse event originated from a 3 cm redness near the CGM application site. No serious adverse events occurred.

## 4. Discussion

Overall, the data presented here demonstrate that the accuracy of the Dexcom G6 sensor was not significantly impacted by different exercise types. While a statistically significant decrease in accuracy (ARD and sensor bias) had been reported for the Dexcom G4 Platinum during moderate aerobic exercise [28], the Dexcom G6 system showed no significant changes in median ARD across aerobic, resistance and HIIT exercise, as illustrated in Figure 1. It was also previously shown that the Dexcom G4 Platinum and Medtronic Paradigm Veo Enlite CGM (Medtronic, Dublin, Ireland) experienced increases in error during continuous and interval training (median ARD for Dexcom/Enlite of 13.34/11.95% at rest and 15.13/14.11% during exercise with median ARD comparison showing a *p*-value of 0.02 for both CGM systems) [44]. Similarly, an increase in negative bias in continuous moderate intensity exercise as opposed to intermittent high-intensity exercise was observed for the previous generation Dexcom G4 Platinum CGM during 90-min cycling sessions [45]. Recently, the FreeStyle Libre CGM (Abbott, Chicago, IL, USA) had also shown significant bias during aerobic exercise with an overall median ARD of 22% [46].

Across most time points, the Dexcom G6 CGM demonstrated a negative bias as compared to the meter, indicating an under-estimation of glucose levels. There is a clear advantage to under-estimating glucose levels as compared to over-estimating glucose levels, as the latter could be a safety concern due to the risk of undetected hypoglycemia. This is particularly true during aerobic exercise where glucose levels can drop rapidly. This CGM under-estimation trend is similar to past observations made on the Medtronic Gold, Dexcom G4 Platinum and Medtronic Paradigm Veo Enlite CGM devices [44,45,47]. As shown in Table 2, the overall negative bias (mean RD) of the Dexcom G6 appeared to be affected by different exercise types, with a significantly greater under-estimation of glucose levels during HIIT when compared to aerobic exercise. Additionally, this negative bias was shown to be significantly impacted as CBG levels became more hyperglycemic and glucose changes more sudden. There was notably an estimated 18- to 19-min lag of the CGM reading during resistance and HIIT exercise, although the estimated lag varied greatly between participants. It is also essential to note that the Dexcom G6 sensor displayed no negative bias when controlling for other variables, with a near-zero mean RD intercept of −0.2% presented in Table 2.

On the other hand, the absolute error showed no significant impact from the different types of exercise, capillary glucose values, time since exercise start, or magnitude of the glucose drop. The observed trend in Table 2 showed slightly lower mean ARD for resistance exercise, followed by HIIT, compared to aerobic exercise. We observed that baseline median ARD was higher in HIIT (16.8%) compared to aerobic and resistance exercise. Since there were no experimental reasons that could explain the higher baseline ARD in HIIT, we believe that this difference is simply due to chance. Mean ARD somewhat displayed worse accuracy with greater glucose drop, but the effect was minimal and not significant.

With mean ARD ranging from 11.2 to 15.9% during aerobic, resistance and HIIT exercises, this study observed somewhat reduced numeric accuracy compared to recent at-rest results of the G6 system in pregnant women with diabetes for example (mean ARD 8.7–11.5%) [33]. Many variables may impact the readings of interstitial glucose during exercise and describing these as errors in accuracy is in part misleading. The differences between the Dexcom G6 and CBG were to some extent explained by lag during the HIIT and resistance exercise sessions, although no significant lag was seen with aerobic exercise. The picture is further complicated as interstitial fluid glucose exhibits a more complex pattern than a simple shifted-in-time blood glucose [22]. Exercise affects the volume and distribution of interstitial fluid, glucose uptake in tissues, and the direction and rate of flow between the vasculature, interstitium and lymphatics [24]. These events likely contribute to a glucose gradient that is further disturbed by a stimulation of endogenous glucose production during exercise [48], and a physiologic lag from the transport of glucose between blood and interstitial compartments [48,49]. As such, there are expected differences between capillary and interstitial glucose to take into account while exercising.

A few limitations to this study should be considered. Although the overall change in glucose followed an expected trend, the magnitude of the glucose drop during exercise (maximum median drop of 2.6 mmol/L) was modest in part likely due to the participants ingesting carbohydrates prior to exercise to prevent hypoglycemia [50,51]. As such, the accuracy of the Dexcom G6 with more rapid declines in glucose remains to be determined. The data demonstrated here did trend towards lower accuracy when glucose levels were dropping more quickly. Moreover, this study only analyzed a small population (n = 24) with limited demographic heterogeneity and a maximum of 6 data points per exercise visits. Additionally, the ICC for exercise sessions was 0.78. This fairly high value indicates that participants in a given session were, to some extent, likely to yield the same outcomes in sensor accuracy. This finding hints towards a potential external effect that was not captured by our data (e.g., time of day, meals, etc.) but seemed to have an influence on the exercise sessions. Similarly, the ICC for participants of 0.43 suggests that sensors may be more accurate for some individuals than others. Another limitation is that the post-exercise window was restricted to 30 min in this study. Biagi et al. had shown a return of CGM accuracy 1 h after exercise cessation in the Medtronic Paradigm Enlite-2 CGM [52], but this could not be assessed here. Similarly, the exercise duration in this study was limited to 20 or 30 min and extending it could potentially yield additional trends. Although using CBG as the glucose reference was practical and clinically relevant, potential variability in meter type and glucose strips may have led to measurement inaccuracies. These results were all collected using the Dexcom G6 CGM and may not be generalizable to other CGM systems. Finally, the in-clinic exercise sessions were conducted using exercise videos and it is unknown if other types of exercise that could be performed in a real-world scenario may have a different impact on the CGM accuracy.

## 5. Conclusions

This study is one of the first comprehensive evaluations of the Dexcom G6 accuracy under different types of exercises. Ultimately, our findings support the use of the Dexcom G6 system to reliably and safely assess glucose levels during 30-min exercise sessions. This is vital information with the recent FDA-approval of Control-IQ, which modulates insulin delivery based on real-time Dexcom G6 CGM data [17].

## Figures and Tables

**Table 1:** Participant demographics and baseline characteristics.

	Mean ± SD (Range)
Age (years)	30.5 ± 6.1 (20.0–41.3)
Weight (kg)	82.4 ± 19.3 (51.8–128.0)
Height (cm)	169.9 ± 17.2 (100.5–188.5)
Sex	10 Male–14 Female
HbA1c (%)	8.8 ± 1.4 (7.1–12.7)
HbA1c (mmol/mol)	73 ± 15.3 (54–115)
Duration of diabetes (years)	15.7 ± 7.0 (3–33)
VO_2_ max (mL/kg/min)	33.8 ± 8.4 (20.4–51.4)
Race—no. (%)	
White	18 (75.0)
Black	4 (16.7)
Other	2 (8.3)

**Table 2:** Mixed-effects linear and gamma regression model summaries for mean relative difference (RD) and mean absolute relative difference (ARD).

	Mean RD (Linear)			Mean ARD (Gamma)		
Measures	Estimate (%)	*p*	95% CI	Estimate (Ratio ^1^)	*p*	95% CI
Intercept	−0.19	0.96	−7.2–6.8	10.85	<0.001	6.97–16.90
Exercise type (vs. aerobic):						
HIIT	−11.3	0.040	−22.0–−0.5	0.980	0.93	0.593–1.617
Resistance	−4.6	0.38	−15.1–5.8	0.933	0.78	0.565–1.541
Reference Meter ^2^	−0.74	0.003	−1.2–−0.3	0.997	0.83	0.971–1.024
Glucose drop ^2^	3.1	<0.001	2.3–3.9	1.035	0.31	0.969–1.105
Time since start	−0.28	0.43	−1.0–0.4	1.027	0.40	0.966–1.092

^1^ Mean ARD estimates are shown as ratios, with the exception of the intercept (%), ^2^ Both meter and glucose drop variables were scaled down by a factor of 10 to facilitate interpretation.

## Data Availability

The de-identified research data for this study are available as a CSV file upon written request and signature of a sharing agreement, as defined by the Oregon Health & Science University Institutional Review Board (OHSU IRB). The data file contains the 272 m and sensor paired values collected during the study along with additional metadata (exercise type, exercise session number, sensor wear time, etc.) necessary to reproduce all results presented here.
